# Supplementary material for: Expression of IL-37 Induces a Regulatory T-Cell-like Phenotype and Function in Jurkat Cells
Source: Cells. 2022 Aug 18;11(16):2565. doi: 10.3390/cells11162565 (PMC9406943; doi:10.3390/cells11162565)
Supplement: Supplementary file 1 [file cells-11-02565-s001.zip › cells-1768677-supplementary.pdf]

Supplemental Figures

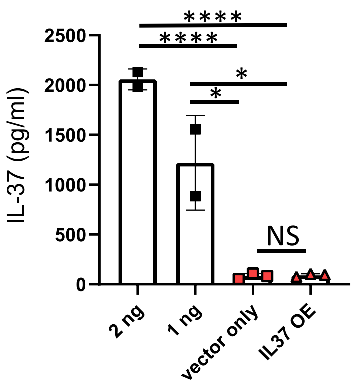

Supplemental Figure S1. IL-37 secretion by *IL37* OE Jurkat cells. ELISA analysis of IL-37 in the supernatant of vector control and *IL37* OE Jurkat cells. Recombinant human IL-37 controls at 2 ng/ml and 1 ng/ml were used as positive controls. Small horizontal lines indicate mean ± s.e.m.. *$P < 0.05$; ****$P < 0.0001$ (Student's t test).

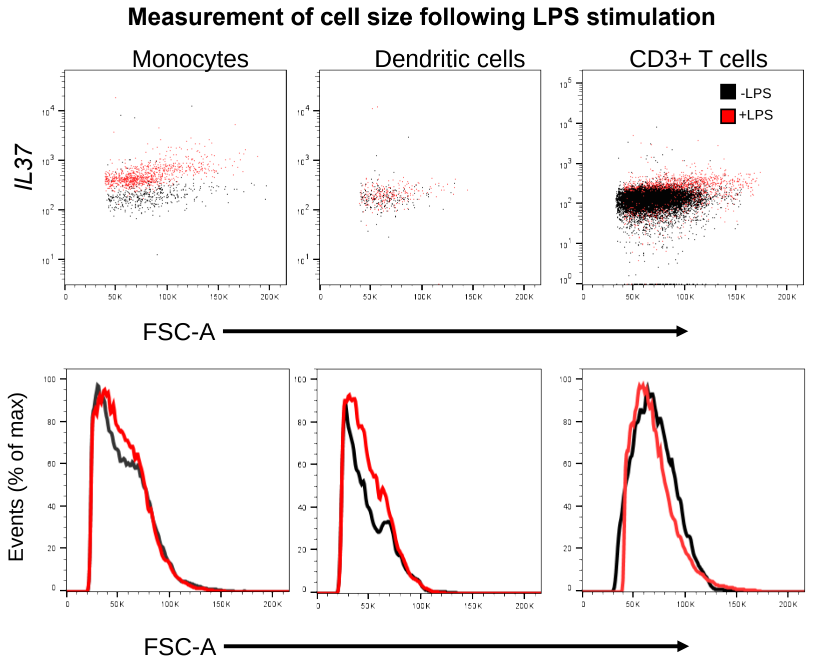

Supplemental Figure S2. LPS treatment resulted in minor alterations in cell size using Primeflow®. Top, monocyte, dendritic and CD3+ T lymphocyte cell size +/-LPS treatment was performed by overlaying density plots of –LPS cells over +LPS cells and observing shifts in the forward scatter. Bottom, monocyte, dendritic and CD3+ T lymphocyte cell size +/-LPS treatment was performed by overlaying histograms of –LPS cells over +LPS cells and observing shifts in the forward scatter histograms.
